# Cross-Host Adaptation of *Campylobacter jejuni* Is Shaped by Chromosomal Backgrounds and Mobile Gene Acquisition, with Human-Associated Traits Emerging Under Limited Mutational Diversification

**DOI:** 10.3390/microorganisms14040874

**Published:** 2026-04-13

**Authors:** Yingdong Li, Zhifeng Ma, Jing Chi, Yinqiu Wang, Minjie Li, Qianru Wang, Lei Lei, Qingliang Chen

**Affiliations:** Department of Microbiological Laboratory, Baoan District Center for Disease Control and Prevention, Shenzhen 518101, China; ylifc@connect.ust.hk (Y.L.);

**Keywords:** *Campylobacter jejuni*, host adaptation, horizontal gene transfer, plasmids, zoonosis, comparative genomics

## Abstract

*Campylobacter jejuni* is a major zoonotic pathogen that circulates among birds, livestock, humans, and environmental reservoirs, yet the genomic mechanisms that enable persistence and transmission across divergent hosts remain incompletely understood. Here, we sequenced 61 *C. jejuni* isolates recovered from multiple host-associated sources in Shenzhen, China, from 2016 to 2023, and analyzed them together with 312 dereplicated publicly available high-quality reference genomes. Phylogenomic analyses resolved three major clades, including one avian-restricted clade and two clades showing frequent cross-host occurrence. Human-associated isolates displayed lower coding density than mammal-associated isolates and significantly higher proteome-level carbon and nitrogen demands than avian-associated isolates. Comparative genomic analyses further revealed strong host-associated divergence in chromosome-encoded, plasmid-encoded, and horizontally acquired gene repertoires. In human-derived isolates, 11 dataset-specific human-unique KEGG genes and 48 human-unique virulence-associated genes were identified, and human-associated strains showed the strongest multidrug-resistance signal across both chromosome-encoded and mobile-gene compartments. Resistance-associated functions enriched in human-associated genomes included antibiotic inactivation, efflux-mediated resistance, target protection/replacement/alteration, reduced permeability, and nutrient-acquisition-associated resistance. By contrast, core host-interaction loci remained under strong purifying selection, indicating that major human-associated traits were linked more closely to mobile gene acquisition than to extensive mutation-driven diversification. Together, these findings support a proposed genome-partition framework of host adaptation in *C. jejuni*, in which relatively stable chromosomal backgrounds are complemented by rapid plasmid- and horizontal-transfer-mediated acquisition of high-impact accessory genes.

## 1. Introduction

*Campylobacter jejuni* is a globally distributed zoonotic pathogen and one of the leading bacterial causes of human gastroenteritis worldwide [1]. It circulates across a wide range of reservoirs, including poultry, cattle, wild birds, companion animals, humans, and environmental sources such as surface water, thereby sustaining repeated zoonotic transmission into human populations [2,3,4]. Although poultry is recognized as the major reservoir for human campylobacteriosis, ruminants, wild birds, and environmental exposure also contribute to infection in a context-dependent manner [5]. Recent studies further indicate that pig- and cattle-associated *Campylobacter* populations may represent important non-poultry reservoirs of antimicrobial-resistant strains, while avian reservoirs beyond broiler chickens, such as turkeys, may also carry isolates with substantial resistance and virulence potential in a One Health context [6,7]. This broad ecological distribution indicates that *C. jejuni* persists across multiple taxonomically and ecologically distinct niches rather than being restricted to a single host-associated lifestyle [8].

This ecological breadth is particularly notable because the hosts colonized by *C. jejuni* differ substantially in intestinal structure, digestive physiology, diet, nutrient availability, and gut microbial community composition [9]. Across vertebrates, host diet and evolutionary history are major determinants of gut microbiome diversity, suggesting that enteric bacteria encounter fundamentally different selective pressures and colonization barriers in different host species [10]. Experimental studies have further shown that resident microbiota can strongly influence *C. jejuni* colonization and dissemination, whereas 16S rRNA-based studies in poultry have mainly characterized caecal community shifts associated with *Campylobacter* colonization [11,12]. Together, these findings imply that successful transmission of *C. jejuni* among animals, humans, and environmental reservoirs requires substantial genomic flexibility to persist and adapt under sharply contrasting ecological conditions [12,13,14].

Despite extensive research, previous studies have largely focused on three areas. First, many investigations have examined source attribution and transmission pathways, particularly along the poultry-associated food chain, using multilocus sequence typing, case-control designs, and related genomic epidemiological approaches [15]. In addition, studies from retail meat systems have shown that chicken- and pig-associated *Campylobacter* populations can be both genetically diverse and antimicrobial-resistant, underscoring the contribution of food chain reservoirs to cross-host dissemination and public-health risk [16]. Second, microbiota-oriented studies have primarily described colonization-associated community shifts or microbiota-mediated resistance [17], rather than the bacterial genomic basis of host switching itself. Third, genomic studies in agricultural and food-production settings have identified traits associated with food chain survival, host-segregating markers, and the emergence of host-specialized lineages, including cattle-adapted lineages [18,19]. However, these studies do not yet explain, within a unified genome-wide framework, how *C. jejuni* rapidly adapts across highly divergent host species and environmental contexts, nor how such adaptive processes contribute specifically to human-associated strains [20,21]. Thus, the genomic mechanisms supporting rapid cross-host dissemination, persistence in distinct gut ecosystems, and adaptation to the human-associated niche remain insufficiently resolved.

In this study, we addressed this gap by sequencing 61 *C. jejuni* isolates recovered from multiple hosts in Shenzhen, China, between 2016 and 2023, and integrating them with all publicly available high-quality genomes. By combining phylogenetic reconstruction with comparative genomic analyses, we sought to characterize host-association patterns and define the genomic features underlying host specificity, cross-host transmission, and adaptation to human-associated strains. In particular, we aimed to define how genomic mutation, plasmid-encoded genes, horizontally acquired genes, and chromosome-encoded genes collectively shape host specificity, cross-host transmission, and adaptation to the human-associated niche.

## 2. Materials and Methods

### 2.1. Sample Collection and Strain Isolation

To investigate *C. jejuni* contamination across different host-associated sources, a total of 831 samples were collected in Shenzhen, Guangdong Province, China, between 2016 and 2023. These included 41 human stool samples, 230 bird meat samples, 250 duck meat samples, and 310 chicken meat samples. A total of 61 *C. jejuni* isolates were subsequently recovered from these samples. Detailed information for all samples is summarized in Table S1. Bacterial isolation of all samples was performed according to GB 4789.9-2014 (Food Microbiological Examination: *C. jejuni*) [22]. Briefly, 25 g of each sample was homogenized in 225 mL of Bolton broth and incubated under microaerobic conditions (85% N_2_, 10% CO_2_, and 5% O_2_) at 36 °C for 4 h, followed by enrichment at 42 °C for 24–48 h. Enriched cultures were streaked onto modified charcoal cefoperazone deoxycholate agar (mCCDA) and Skirrow agar and incubated microaerobically at 42 °C for 24 to 48 h. Presumptive *Campylobacter* colonies were purified on Columbia blood agar plates. Biochemically confirmed pure isolates were used for genomic DNA extraction and whole-genome sequencing.

### 2.2. Genomic DNA Extraction and Genome Sequencing

Genomic DNA was extracted from bacterial colonies cultured on selective agar plates using the Genomic DNA Extraction Kit (Tiangen Biotech Co., Ltd., Beijing, China; catalog no. DP302-02) according to the manufacturer’s instructions. DNA quality and quantity were preliminarily assessed by agarose gel electrophoresis and NanoDrop spectrophotometry, respectively. The extracted DNA was further subjected to PCR amplification using *Campylobacter jejuni*-specific primers for preliminary identification [23]. The remaining DNA was stored at −20 °C and sent to Novogene Co., Ltd. (Beijing, China) for library preparation and high-throughput sequencing. Paired-end sequencing (PE150) was performed on the Illumina NovaSeq 6000 platform (Illumina, San Diego, CA, USA).

### 2.3. Genome Assembly and Refinement

Sequencing reads were assigned to individual samples according to their barcode sequences. Clean 150-bp paired-end reads were generated by removing adapters, barcodes, reads containing poly-N, and low-quality reads from the raw sequencing data. The filtered reads were quality-checked using FastQC (Babraham Bioinformatics) and assembled using MEGAHIT v1.2.9 with the parameters --k-min 27 --k-max 147 --k-step 12 [24]. Genome completeness and contamination were assessed using CheckM v1.1.2 [25], and taxonomic classification was performed using GTDB-Tk v1.6.0 [26].

Reference genomes with completeness >80% and contamination <1% were downloaded from the BV-BRC database together with their isolation metadata for subsequent phylogenetic and comparative genomic analyses. Redundant genomes were removed by de-replication using dRep v1.0.3 with the dereplicate workflow (primary ANI cutoff, 0.90; secondary ANI cutoff, 0.99; secondary comparison algorithm, ANIn; minimum alignment coverage threshold, 0.10; clustering algorithm, average) [27]. Information for the representative reference genomes for downstream analysis is summarized in Table S2.

### 2.4. Phylogenomic Tree Construction

Phylogenomic trees were inferred for the de-replicated genome set using two complementary approaches. First, a marker-gene phylogeny was constructed based on the GTDB-Tk bacterial marker gene set (bac120). Specifically, GTDB-Tk v1.6.0 was used to identify and align the 120 conserved bacterial marker genes from each genome, followed by concatenation of the marker-gene alignments using the GTDB-Tk workflow. Second, an ortholog-based phylogeny was constructed using single-copy orthologs across all genomes. OrthoFinder v2.5.5 was used to infer orthogroups and identify single-copy orthologs shared among genomes. The single-copy orthologs were concatenated to generate a supermatrix for phylogenomic inference. For both approaches, maximum-likelihood phylogenies were inferred using IQ-TREE 2 with automatic model selection enabled by ModelFinder (-m MFP), and branch support was assessed using 1000 ultrafast bootstrap replicates (-B 1000).

### 2.5. Identification of Plasmid-Encoded Genes and Horizontally Acquired Genes

Putative horizontally acquired genes were first identified from assembled contigs using WAAFLE (Workflow to Annotate Assemblies and Find LGT Events), which detects candidate lateral gene transfer regions on the basis of sequence homology patterns and taxonomic provenance [28]. Genes located within WAAFLE-predicted transfer regions were therefore defined as horizontally acquired genes. To provide an independent validation of these predictions, all predicted protein-coding sequences were further analyzed using HGTector. Briefly, protein sequences were searched against a local reference protein database using DIAMOND/BLASTp (v2.1.24) following the recommended HGTector workflow, and hits were filtered according to alignment quality and taxonomic criteria [29]. HGTector then partitioned homologous hits into self, close, and distal taxonomic groups and calculated weighted scores based on normalized bit scores to identify candidate HGT-derived genes. The search step was performed using the commonly reported thresholds of --maxhits 500, --evalue 1 × 10^−20^, --identity 50, and --coverage 50, whereas the analysis step used weighted scoring, z-score-based outlier removal, automatic bandwidth optimization, and a silhouette cutoff of 0.5. Genes supported by both WAAFLE and HGTector were considered high-confidence horizontally acquired genes. Putative plasmid contigs were identified from the same assemblies using PLASMe (v1.1) with default settings, and genes encoded on these contigs were defined as plasmid-encoded genes [30]. Coding sequences on predicted plasmid contigs were identified using Prodigal v2.6.3 with the -p single parameter for downstream functional annotation [31]. When a gene was simultaneously located on a PLASMe-predicted plasmid contig and within a WAAFLE/HGTector-supported transfer region, it was classified as a plasmid-associated gene. For downstream compartment-resolved analyses, such genes were retained within the plasmid-gene compartment and were not double-counted as independent chromosome-derived HGT events.

### 2.6. Gene Prediction and Functional Annotation

Protein-coding sequences (CDSs) were predicted for each genome using Prodigal v2.6.3 with the -p single parameter [31]. The predicted CDSs, together with the horizontally acquired genes and plasmid-encoded genes identified above, were annotated against the Kyoto Encyclopedia of Genes and Genomes (KEGG) database [32] and the Transporter Classification Database (TCDB; TransportDB v2.0) [33] using DIAMOND v2.0.4 [34], with a minimum alignment coverage of 75% and an e-value threshold of 1 × 10^−10^. KEGG pathway reconstruction was performed using the “Reconstruct Pathway” module in KEGG Mapper. Assembly-related genomic features, including genome size, gene number, coding density, and GC content, were calculated using CheckM v1.1.2 [25]. The estimated genome size was adjusted as follows: estimated genome size = genome size/(completeness + contamination) [35].

### 2.7. Genetic Variation and Elemental Composition Metrics

Population genetic statistics and nucleotide variation metrics, including the ratio of non-synonymous to synonymous substitutions (*pN*/*pS*), were calculated at both the genome (based on homologous genes identified across genomes) and gene levels using KaKs_Calculator 3.0 [36]. The carbon atoms per amino acid residue side chain (C-ARSC) and nitrogen atoms per amino acid residue side chain (N-ARSC) were calculated using custom scripts from a publicly available repository [37].

### 2.8. Statistical Analysis

Statistical analyses were performed in R (version 4.3.1). Prior to between-group comparisons, the distribution of continuous variables was assessed using the Shapiro–Wilk test, and homogeneity of variance was evaluated using Levene’s test. Because several variables did not satisfy assumptions of normality and/or homoscedasticity, non-parametric tests were used for group comparisons. Differences among host groups were first evaluated using the Kruskal–Wallis test, followed by pairwise Wilcoxon rank-sum tests for post hoc comparisons where appropriate. To control the false discovery rate, *p*-values from multiple comparisons were adjusted using the Benjamini–Hochberg procedure. Adjusted *p*-values (*q* values) < 0.05 were considered statistically significant. Effect sizes were reported as rank-biserial correlation for non-parametric comparisons. Given the unequal sample sizes across host groups, approximate post hoc power analyses were additionally performed as heuristic indicators of support strength, and non-significant results were interpreted cautiously.

Functional enrichment analyses for KEGG and VFDB categories were performed using hypergeometric tests, with the annotated background gene set used as the reference universe. For each category, odds ratios, 95% confidence intervals, and Benjamini–Hochberg-adjusted *q* values were calculated. To assess potential confounding by sampling heterogeneity, mixed-effects models were additionally fitted for selected response variables using host as a fixed effect and geographic region and isolation year as random effects, where metadata completeness allowed. Sensitivity analyses based on metadata-complete subsets were also performed where necessary.

### 2.9. Data Availability Statement

The genomes described in this study are deposited in NCBI under BioProject accession PRJNA1298557. Scripts and reference-genome metadata are available at the project GitHub repository: https://github.com/ylifc/Campylobacter_jejuni (accessed on 9 March 2026).

## 3. Results

### 3.1. The Phylogenomic Relationships of C. jejuni Genomes

**Figure 1:**
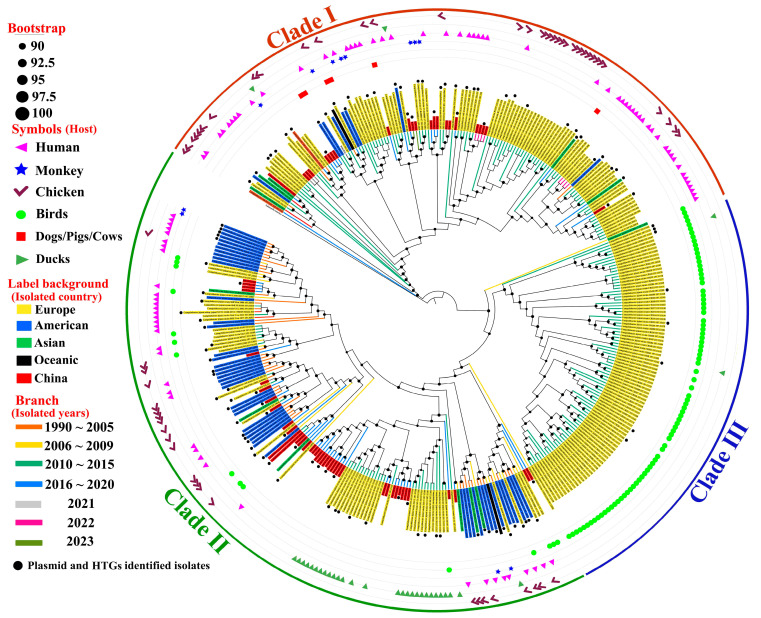
Phylogenomic relationships of *Campylobacter jejuni* genomes across host sources and geographic regions. Maximum-likelihood phylogeny of dereplicated *C. jejuni* genomes inferred from the GTDB-Tk bac120 marker set. Outer colored arcs delineate the three major phylogenomic clades (Clades I–III). Branch colors represent isolation year, whereas the background colors of terminal labels indicate geographic origin (Europe, America, Asia, Oceania, or China). Symbols positioned outside the tree denote the host source. Black circles at nodes indicate bootstrap support values scaled from 90 to 100. Black dots mark isolates in which plasmids and horizontally transferred genes (HTGs) were validated by both approaches (WAAFLE and HGTector).

Among the 831 samples analyzed, 61 *Campylobacter jejuni* isolates were successfully recovered and whole-genome sequenced, including 21 isolates from bird meat, 16 from chicken meat, 23 from duck meat, and 1 from human stool. All samples were collected from markets in Shenzhen, China. All assemblies were of high quality, with estimated completeness ranging from 97.10% to 99.66% and contamination consistently below 1% (Table 1). To resolve phylogenomic relationships among these isolates, maximum-likelihood phylogenies of all 373 genomes (312 from quality-filtered and de-replicated reference genomes; 61 from this study) were reconstructed using two complementary strategies: (i) a concatenated alignment of 120 conserved single-copy bacterial marker genes (Figure 1) and (ii) a concatenated alignment of single-copy orthologs inferred by OrthoFinder (Figure S1). Both approaches consistently resolved three major clades. Two clades contained isolates recovered from multiple host types, indicating frequent cross-host occurrence, whereas the remaining clade was composed exclusively of avian-associated isolates, suggesting strong host restriction to birds, including ducks.

Within the three clades, the lineage designated as clade I occupied the deepest branching position in both phylogenies. Notably, isolate P4-122C_S20 (chicken-derived, China, 2017) was placed near the basal node of clade I, indicating an early-diverging position among the genomes analyzed in this study (Figure 1 and Figure S1). Among the genomes analyzed in this study, the earliest human-associated isolate was OXC6395, recovered in Europe in 2010, whereas the earliest human-associated isolate from China was 21CJ13, recovered in 2016.

### 3.2. Genomic Features of C. jejuni Genomes from Different Hosts

**Figure 2:**
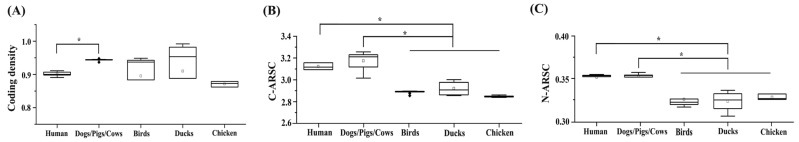
Host-associated genomic characteristics of *Campylobacter jejuni*. Comparisons of (**A**) coding density, (**B**) carbon atoms per amino acid residue side chain (C-ARSC), and (**C**) nitrogen atoms per amino acid residue side chain (N-ARSC) among *C. jejuni* genomes derived from humans, mammals (dogs, pigs, and cattle), birds, ducks, and chickens. Asterisks (*) indicate significant pairwise differences after Benjamini–Hochberg correction. Corresponding significant robustness contrasts after controlling for geographic region and isolation year are summarized in Table S3.

Comparative analyses across all *C. jejuni* isolates revealed host-associated differences in coding density and proteome elemental composition, particularly in carbon atoms per amino acid residue side chain (C-ARSC) and nitrogen atoms per amino acid residue side chain (N-ARSC) (Figure 2). Post hoc comparisons showed that human-associated isolates had a lower coding density than mammal-associated isolates (dogs, pigs, and cattle) after Benjamini–Hochberg correction. In the metadata-complete subset, this difference remained significant after controlling for geographic region and isolation year in mixed-effects models (Table S3). Moreover, human- and mammal-associated isolates exhibited higher C-ARSC and N-ARSC values than avian (birds, ducks, and chickens)-associated isolates after Benjamini–Hochberg correction. These differences also remained significant in the metadata-complete subset after adjustment for geographic region and isolation year, both for the human-versus-avian contrast (Table S3) and for the mammal-versus-avian contrast (Table S3), indicating that the principal host-associated pattern was robust to geographic and temporal sampling structure. Chicken-derived isolates exhibited the narrowest variation in both coding density and elemental composition. Overall, the distributions of C-ARSC and N-ARSC were broadly similar across avian-associated groups, whereas human- and mammal-associated isolates showed partially overlapping but generally elevated values.

### 3.3. Host-Associated Functional Repertoires Revealed by KEGG and VFDB Annotation

**Figure 3:**
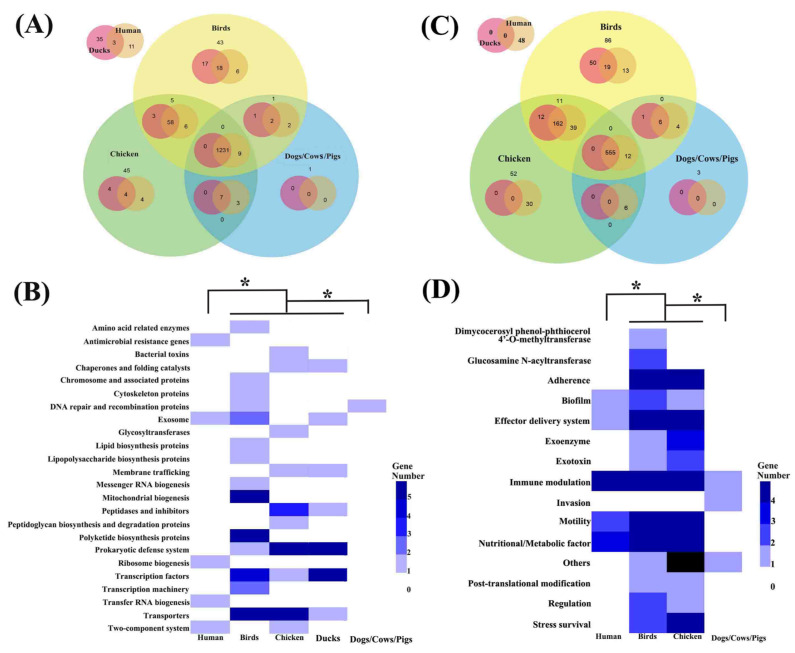
Host-associated divergence in KEGG- and VFDB-annotated functional repertoires. (**A**) Overlap and uniqueness of KEGG-annotated genes among host groups. (**B**) Numbers of host-unique KEGG genes partitioned by major functional modules. The functional composition of genes associated with human- and mammal-associated isolates differed significantly from that of avian-associated isolates after Benjamini–Hochberg correction. Corresponding robustness analyses controlling for geographic region and isolation year are summarized in Table S3. (**C**) Overlap and uniqueness of VFDB-annotated virulence-associated genes among host groups. (**D**) Numbers of host-unique virulence-associated genes partitioned by functional class. Asterisks (*) indicate significant pairwise differences after Benjamini–Hochberg correction. Corresponding significant robustness contrasts after controlling for geographic region and isolation year are summarized in Table S3.

Functional annotation based on KEGG revealed pronounced host-associated differences in gene content among *C. jejuni* isolates (Figure 3A). The number of host-unique genes was 11 in human-derived isolates, 35 in duck-associated isolates, 43 in bird-associated isolates, 45 in chicken-associated isolates, and 1 in mammal-associated isolates (dogs, cattle, and pigs). Hypergeometric enrichment analysis of KEGG categories further identified distinct host-associated functional patterns after accounting for background gene counts and group sizes (Figure 3B; Table S3). The functional composition of genes associated with human- and mammal-associated isolates differed significantly from that of avian-associated isolates after Benjamini–Hochberg correction. In the metadata-complete subset, these host-associated patterns remained significant after adjustment for geographic region and isolation year, indicating that the major enrichment signals were robust to sampling heterogeneity. Specifically, human-associated genes were enriched in antimicrobial resistance, membrane-associated processes, ribosome biogenesis, tRNA-related processes, and two-component signal transduction. Bird-associated genes were enriched in categories related to secondary metabolite biosynthesis, transcription-associated functions, and transporter systems. Chicken-associated genes were mainly assigned to peptidases and inhibitors, prokaryotic defense systems, and transporter functions, whereas the mammal-unique gene was annotated as being involved in DNA repair and recombination.

Virulence-factor annotation using VFDB also revealed pronounced host-associated differences in virulence gene repertoires among *C. jejuni* isolates (Figure 3C,D). Hypergeometric enrichment analysis of VFDB categories identified distinct host-linked virulence patterns after accounting for background gene counts and group sizes. The functional composition of virulence-associated genes in human- and mammal-associated isolates differed significantly from that of avian-associated isolates after Benjamini–Hochberg correction. In the metadata-complete subset, these virulence-associated patterns remained significant after adjustment for geographic region and isolation year, indicating that the major signals were robust to sampling heterogeneity (Table S3). Human-derived isolates contained 48 human-unique virulence-associated genes, whereas no duck-unique virulence-associated genes were detected in duck-derived isolates; bird-, chicken-, and mammal-derived isolates contained 86, 52, and 3 unique virulence-associated genes, respectively. Human-associated virulence genes were mainly related to immune modulation, motility, and nutritional or metabolic functions. Bird-associated virulence genes were enriched in adherence, effector delivery systems, immune modulation, motility, and nutritional or metabolic factors. Chicken-associated virulence genes were predominantly assigned to adherence, effector delivery systems, immune modulation, motility, nutritional or metabolic factors, and stress survival. Because only three mammal-unique virulence-associated genes were detected in mammal-derived isolates, their functional interpretation should be treated cautiously.

### 3.4. Host-Associated Chromosome-Encoded, Plasmid-Encoded, and Horizontally Acquired Functional Genes

**Figure 4:**
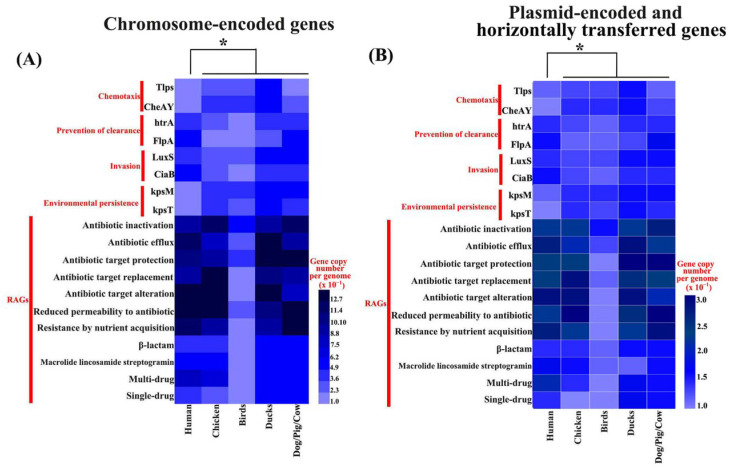
Host-associated distribution of key functional genes across genomic compartments in *Campylobacter jejuni*. Heatmaps showing the mean copy number per genome of representative loci involved in chemotaxis, prevention of clearance, host-cell invasion, environmental persistence, and antimicrobial resistance across different host-associated groups. Analyses are shown separately for (**A**) chromosome-encoded genes and (**B**) plasmid-encoded and horizontally transferred genes. Columns correspond to host groups (human, chicken, birds, ducks, and dogs/pigs/cattle), whereas rows correspond to individual genes or antimicrobial resistance-associated gene categories. Color gradients indicate copy number per genome (×10^−1^). RAGs, resistance-associated genes. Asterisks (*) indicate statistically significant host-associated differences after Benjamini–Hochberg correction. Corresponding significant robustness contrasts after controlling for geographic region and isolation year are summarized in Table S3.

To compare host-associated functional potentials across genomic compartments, we profiled the copy numbers of key loci involved in chemotaxis, prevention of clearance, host-cell invasion, environmental persistence, and antimicrobial resistance across isolates from different host sources (Figure 4A,B). For chromosome-encoded loci (Figure 4A), host-linked differences were evident across multiple functional categories. Hypergeometric enrichment analysis of chromosome-encoded loci further identified distinct host-associated functional patterns after accounting for background gene counts and group sizes. The functional composition of chromosome-encoded loci in human-associated isolates differed significantly from that of other host-associated groups after Benjamini–Hochberg correction. In the metadata-complete subset, these chromosome-encoded patterns remained significant after adjustment for geographic region and isolation year, indicating that the major signals were robust to sampling heterogeneity (Table S3). Specifically, human-associated isolates showed higher copy numbers of loci related to prevention of clearance and host-cell invasion, whereas avian-associated isolates, particularly chicken- and bird-derived isolates, displayed stronger representation of chemotaxis- and environmental persistence-associated loci. Host-associated differences were also apparent in antimicrobial resistance-related gene categories. Across chromosome-encoded content, non-avian-associated isolates showed higher overall representation of resistance mechanisms, including antibiotic inactivation, efflux-mediated resistance, target protection/replacement/alteration, reduced permeability, and nutrient acquisition-associated resistance (Figure 4A). Notably, human-associated isolates exhibited the strongest multidrug-resistance signal among all host groups.

Patterns derived from plasmid-encoded genes and horizontally transferred genes (HTGs) were broadly consistent with those observed for chromosome-encoded loci (Figure 4B). Hypergeometric enrichment analysis further identified distinct host-associated functional patterns among these mobile genetic elements after accounting for background gene counts and group sizes. The functional composition of plasmid-encoded and HTG-associated loci in human-associated isolates differed significantly from that of other host-associated groups after Benjamini–Hochberg correction, and these differences remained robust after controlling for geographic region and isolation year in the metadata-complete subset (Table S3). In particular, resistance-related gene categories were more abundant in human-associated isolates, supporting a major role for mobile genetic elements in the enrichment of antimicrobial resistance determinants in strains associated with human infection.

### 3.5. Evolutionary Signatures of Key Functional Genes Across Host-Associated Isolates

**Figure 5:**
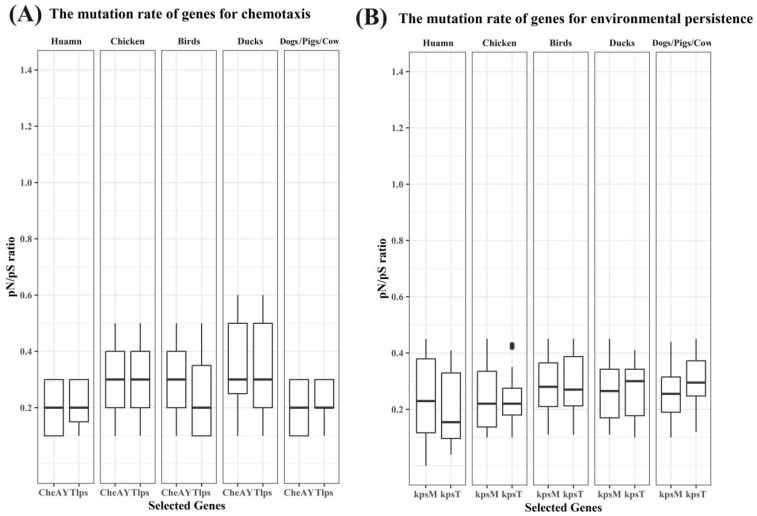
Host-structured *pN*/*pS* patterns of chemotaxis- and persistence-associated genes. Gene-wise *pN*/*pS* distributions for representative loci involved in chemotaxis and environmental persistence.

**Figure 6:**
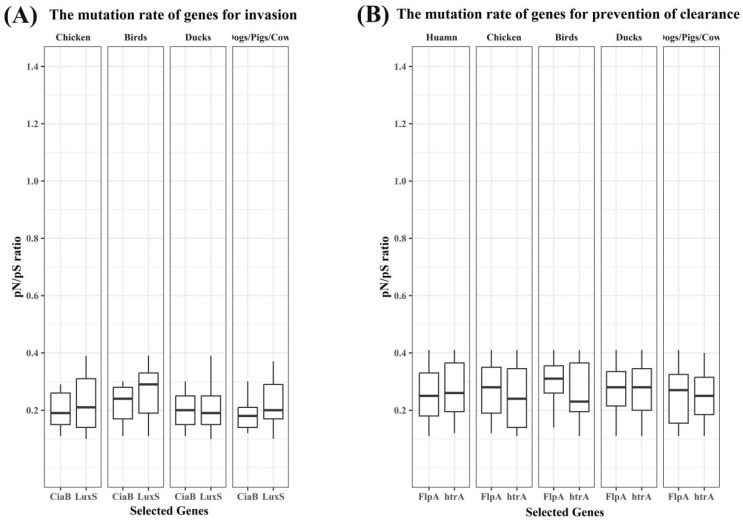
Strong purifying selection on invasion- and clearance-associated genes across host groups. Gene-wise *pN*/*pS* distributions for representative loci involved in host-cell invasion and prevention of clearance.

To assess selective constraints on host-relevant functions, we compared *pN*/*pS* ratios for representative genes involved in chemotaxis, environmental persistence, host-cell invasion, and prevention of clearance across isolates from different host sources (Figure 5 and Figure 6). Overall, *pN*/*pS* ratios for these loci remained well below 1 across host groups, consistent with predominant purifying selection, but the magnitude and dispersion differed among functional categories and hosts.

For chemotaxis-associated genes (*CheA*, *CheY*, and *Tlps*), avian-associated isolates, particularly those from chickens and ducks, displayed broader *pN*/*pS* distributions than human-associated isolates (Figure 5A). Among avian groups, duck-associated isolates showed the widest dispersion for chemotaxis loci, indicating greater heterogeneity in evolutionary constraints across strains (Figure 5A). In contrast, human- and mammal-associated isolates generally exhibited narrower *pN*/*pS* ranges for chemotaxis genes (Figure 5A).

Environmental persistence-related genes (*kpsM* and *kpsT*) showed consistently higher *pN*/*pS* levels than invasion- or clearance-related genes and exhibited clear host-associated differences (Figure 5B). In particular, avian-associated isolates (birds and ducks) and mammal-associated isolates tended to show higher central *pN*/*pS* values for *kpsM*/*kpsT* than human-associated isolates, with *kpsT* in humans showing a comparatively lower distribution (Figure 5B). Together, these patterns indicate that environmental persistence loci display stronger host-linked variation in evolutionary signatures than several other functional categories assessed here.

In contrast, genes associated with host-cell invasion (*ciaB* and *luxS*) and prevention of clearance (*flpA* and *htrA*) exhibited uniformly low *pN*/*pS* values with relatively limited dispersion across host groups (Figure 6A,B). This pattern was consistent across avian-, mammal-, and human-associated isolates (Figure 6), indicating strong and broadly conserved selective constraints on these loci across host contexts.

## 4. Discussion

Our phylogenomic reconstruction, integrating newly sequenced Shenzhen isolates with 312 dereplicated publicly available genomes, resolved three major clades with contrasting host-association patterns, including one lineage largely restricted to avian hosts and two lineages detected across multiple host sources. This structure is consistent with the long-recognized role of poultry and other birds as major reservoirs for *C. jejuni* and major contributors to human infection [1,38]. This avian reservoir should not be viewed as limited to broiler chickens alone, as recent One Health-oriented work has shown that turkeys may also harbor *Campylobacter* isolates with substantial antimicrobial-resistance and virulence potential, broadening the avian host spectrum relevant to transmission [7]. The strong avian restriction observed for one lineage also agrees with previous population studies showing marked host specificity among wild-bird-associated *C. jejuni* populations, in which host species can be a major determinant of genotype composition [39,40]. By contrast, the other two clades showed broader host occurrence, including isolates recovered from humans and domestic animals, which is consistent with prior evidence that host-generalist *C. jejuni* populations can switch hosts frequently enough to erode simple host-association signals [41]. This pattern also aligns with the concept of “cryptic ecology,” whereby lineages that appear broadly distributed may still occupy distinct ecological niches shaped by livestock production systems, contact structure, and exposure opportunities [42]. This interpretation is also consistent with evidence from Asian retail meat systems showing that chicken- and pig-associated *Campylobacter* populations can display substantial genotypic diversity together with antimicrobial resistance, thereby providing repeated opportunities for food chain dissemination and cross-host exposure [16].

At the local scale, the Shenzhen isolates were interspersed among genomes sampled from multiple regions rather than forming an exclusive monophyletic cluster, indicating that local diversity likely reflects both locally circulating lineages and lineages connected to broader transmission networks. This is epidemiologically relevant as the coexistence of a host-restricted avian lineage and host-generalist lineages provides an informative evolutionary contrast for mechanistic inference. In this context, our results support a proposed genome-partition framework of host adaptation in which chromosome-encoded backgrounds provide a relatively stable evolutionary scaffold, whereas plasmids and other horizontally acquired genes act as more rapidly exchangeable modules that expand ecological breadth, facilitate host switching, and contribute disproportionately to the emergence of human-associated traits.

We also observed pronounced host-associated shifts in proteome-level elemental composition, quantified as carbon atoms per amino acid residue side chain (C-ARSC) and nitrogen atoms per amino acid residue side chain (N-ARSC), together with differences in coding density. Because C-ARSC and N-ARSC were calculated across the predicted whole proteome of each genome, these shifts reflect genome-scale compositional divergence rather than changes confined to a small number of loci [43,44]. Human- and mammal-associated isolates exhibited higher C-ARSC and N-ARSC values than avian-associated isolates, whereas chicken-associated isolates showed comparatively constrained variation in coding density and elemental composition. These host-linked differences are biologically plausible because vertebrate gut habitats differ not only in microbial community structure but also in nutrient landscape, digestive physiology, transit time, and temperature regime. In this context, the elevated C-ARSC and N-ARSC values in human- and mammal-associated isolates may reflect adaptation to mammalian gut environments with distinct resource inputs and competitive constraints relative to avian hosts. Conversely, the narrower distributions observed in chicken-associated isolates may be consistent with a more constrained ecological regime in this reservoir. Although these proteome-level metrics do not identify a single causal mechanism, they support the idea that *C. jejuni* lineages associated with different hosts encode distinct resource-demand strategies shaped by long-term ecological differences among host environments [9,19,45].

Our KEGG- and VFDB-based analyses, together with the compartment-resolved gene comparisons, indicate that host-associated adaptation in *C. jejuni* is structured across both stable and flexible genomic layers. The chromosome-encoded component already shows clear host-linked biases in the five host-relevant modules examined here, namely, chemotaxis, prevention of clearance, host-cell invasion, environmental persistence, and antimicrobial resistance. For avian-associated isolates, elevated signals in chemotaxis loci such as *cheA*, *cheY*, and transducer-like proteins are biologically coherent because chemotaxis contributes centrally to navigation of intestinal gradients and colonization in animal models [46,47,48,49,50]. Likewise, the enrichment of persistence-related loci such as *kpsM* and *kpsT* is notable because capsule-associated gene clusters contribute to serum resistance, persistence, and virulence phenotypes in *C. jejuni* [51]. By contrast, in human-associated isolates, enrichment of invasion- and clearance-related functions is consistent with the established roles of *ciaB* in epithelial invasion, *flpA* in fibronectin-mediated adhesion, and *htrA* in epithelial barrier disruption [52,53,54]. The inclusion of *luxS* within invasion-associated modules is also relevant because *LuxS*/AI-2-linked signaling and metabolism have been implicated in colonization and virulence-associated phenotypes in *C. jejuni* [55]. Together, these host-linked chromosome-level differences suggest that host association is underpinned not only by transient gene gain but also by relatively stable functional backbones shaped by long-term retention and loss across lineages.

Mobile genetic elements appear to amplify these host-linked configurations by disproportionately enriching certain traits, particularly antimicrobial resistance in human-associated strains. In our data, resistance-associated categories were elevated not only among chromosome-encoded genes but also among plasmid-encoded and horizontally acquired genes, and the strongest multidrug-resistance signal was consistently observed in human-associated isolates. This is consistent with well-established mechanisms of *C. jejuni* resistance that combine chromosomal determinants, such as the *CmeABC* multidrug efflux pump, with horizontally transferable resistance genes [56,57]. Conjugative tetracycline-resistance plasmids of the *pTet* family, which frequently carry tet(O), provide a canonical example of how plasmids can rapidly import clinically relevant resistance modules and disseminate them by conjugation [58,59]. Plasmids may also contribute virulence-associated traits; for example, the *pVir* plasmid has been linked to invasion-related phenotypes and more severe clinical outcomes, illustrating how plasmid acquisition can modify host interaction potential [60,61]. This broader interpretation is supported by recent surveillance studies showing that both mammalian reservoirs, such as pigs and cattle, and underappreciated avian reservoirs, such as turkeys, can harbor *Campylobacter* populations with substantial antimicrobial-resistance and virulence potential. Together with our results, these studies reinforce a One Health view in which multiple livestock-associated reservoirs contribute to the circulation and amplification of clinically relevant *Campylobacter* lineages [6,7]. Accordingly, the concordance between chromosome-level patterns and mobile-gene patterns supports a model in which stable chromosomal backgrounds define longer-term host-associated functional tendencies, whereas plasmids and horizontal gene transfer accelerate ecological adjustment by importing high-impact accessory modules.

At the same time, our results also show that not all host-associated differences should be interpreted in the same way. In particular, the number of strictly host-unique genes was small for some groups, especially mammal-associated isolates. For this reason, we distinguish between host-unique genes, which in the present study refers strictly to genes detected only within one host group in this dataset and broader host-associated patterns, which are supported by enrichment analyses, copy-number differences, and robustness analyses. The sparse unique-gene sets should therefore be interpreted cautiously as cohort-level observations rather than definitive host-specific markers. The broader biological signal in our data comes not from a single small set of unique genes but from recurrent host-linked shifts in functional composition across the chromosome-encoded, plasmid-encoded, and horizontally acquired compartments.

The selective-pressure analyses provide a complementary perspective on these host-associated patterns. Across host sources, invasion- and clearance-related loci such as *ciaB*, *flpA*, *htrA*, and *luxS* showed uniformly low and relatively narrow *pN*/*pS* distributions, indicating strong purifying selection and supporting the existence of a conserved host-interaction core maintained during circulation across divergent hosts. This is consistent with experimental evidence that these genes occupy key bottlenecks in colonization and disease, including epithelial invasion, fibronectin-mediated adhesion, and epithelial barrier disruption [53,54,62]. By contrast, chemotaxis- and environmental persistence-related loci displayed broader, host-structured *pN*/*pS* distributions, suggesting that sensing/navigation and persistence modules experience more heterogeneous selective regimes across host environments. In particular, the relatively higher and broader *pN*/*pS* distributions of *kpsM* and *kpsT* in avian-associated isolates may reflect relaxed selective constraint and/or more heterogeneous adaptive pressures in avian reservoirs; our current data do not allow these alternatives to be separated definitively, but they do indicate that persistence-related loci experience more variable host-linked evolutionary regimes than the more conserved invasion and clearance genes.

The focal host-interaction loci examined here exhibited uniformly low *pN*/*pS* values across host groups, indicating strong purifying selection and substantial functional constraint. This pattern is consistent with the view that major host-associated differences in the present dataset are associated more strongly with accessory-gene remodeling through HTG and plasmid acquisition than with extensive amino-acid-changing diversification in these core loci. Such an interpretation does not exclude a role for mutation elsewhere in the genome, but it does suggest that for the host-relevant traits examined here, mobile gene acquisition constitutes the more prominent adaptive route.

Our findings also have ecological and epidemiological implications. The coexistence of host-restricted avian lineages and host-generalist lineages suggests that *C. jejuni* transmission into humans is shaped by both stable reservoir structure and dynamic gene exchange. Avian reservoirs, especially poultry-associated systems, likely provide repeated opportunities for amplification and dissemination, whereas generalist lineages carrying mobile resistance or virulence-associated modules may be especially well positioned to cross host boundaries. In this sense, host adaptation in *C. jejuni* should not be viewed solely as specialization to a single reservoir but also as the emergence of genomic configurations that facilitate persistence across multiple interconnected niches. This perspective is consistent with a One Health view of *Campylobacter* evolution, in which animal reservoirs, food production systems, human exposure, and mobile genetic elements jointly shape the emergence of clinically relevant strains.

Several limitations should also be acknowledged. First, although the inclusion of 312 dereplicated public reference genomes substantially broadened the comparative evolutionary context of the analysis, publicly available genomes vary in metadata completeness, sampling design, and geographic/temporal resolution relative to the newly sequenced Shenzhen isolates. As a result, some residual heterogeneity may remain even after mixed-effects sensitivity analyses on metadata-complete subsets. Second, the number of strictly host-unique genes was small for some host groups, particularly mammal-associated isolates, and these genes should therefore be interpreted cautiously as dataset-specific observations rather than universal host-specific markers. Third, the present study is comparative and inferential in nature; direct experimental validation will be required to confirm the mechanistic roles of specific plasmids, horizontally acquired genes, and candidate host-associated loci in cross-host adaptation and transmission. Despite these limitations, the integration of newly sequenced Shenzhen isolates with a broad public reference set, together with compartment-resolved genomic analyses, provides a coherent framework for understanding how stable chromosomal backgrounds and rapidly acquired mobile genes jointly shape host association, host switching, and the emergence of human-associated pathogenic traits in *C. jejuni*.

## Figures and Tables

**Table 1:** Assembled genome statistics.

Genomes	Completeness	Contamination	Number of ORFs
WQ2018012	99.49	0.29	1749
BWQ2018034	99.66	0.21	1631
BWQ2018033	99.32	0.15	1753
BWQ2018032	99.4	0.11	1611
BWQ2018031	99.4	0.15	1801
BWQ2018030	99.57	0.11	1837
BWQ2018029	99.4	0.11	1677
BWQ2018028	99.37	0.11	1830
BWQ2018027	99.32	0.26	1784
BWQ2018026	99.32	0.26	1699
BWQ2018025	97.11	0.15	1649
BWQ2018024	97.11	0.15	1612
BWQ2018023	99.57	0.11	1662
BWQ2018022	99.66	0.24	1839
BWQ2018021	99.57	0.11	1626
BWQ2018020	99.36	0.11	1693
BWQ2018019	99.57	0.11	1688
BWQ2018017	99.17	0.11	1763
BWQ2018016	99.44	0.19	1775
BWQ2018015	99.53	0.11	1623
BWQ2018014	99.57	0.18	1711
BWQ2018013	86.76	0.18	1754
BWQ2018012	99.49	0.29	1673
BWQ2018010	99.57	0.15	1788
BWQ2018009	99.4	0.15	1689
BWQ2018008	99.38	0.11	1841
BWQ2018007	99.57	0.11	1708
BWQ2018006	99.29	0.11	1714
BWQ2018005	99.38	0.15	1625
BWQ2018003	99.4	0.11	1650
BWQ2018002	99.44	0.22	1648
BWQ2018001	99.34	0.11	1842
BWQ2017004	99.57	0.16	1814
BWQ2017003	99.57	0.16	1745
BWQ2017002	99.57	0.16	1798
BWQ2017001	99.57	0.16	1694
BWQ2016002	99.29	0.26	1721
BWQ2016001	99.38	0.11	1741
B2023021	94.56	0	1675
B2023023	99.37	0.11	1656
B2022033	99.37	0.11	1786
B2022032	99.37	0.11	1712
B2022031	99.4	0.19	1663
B2022030	99.38	0.26	1824
B2021152	97.1	0.28	1840
B2021019	99.4	0.19	1677
B2021018	99.38	0.26	1823
B2021017	99.12	0.33	1684
B2021024	99.44	0.33	1629
B2021015	99.44	0.33	1825
B2019099	99.44	0.33	1622
B2019098	99.44	0.2	1826
B2019097	99.44	0.11	1843
B2019096	99.44	0	1765
B2019044	100	0.11	1844
B2019043	99.44	0	1832
B2019029	100	0.11	1821
B2019028	100	0	1811
B2019027	100	0.11	1741
B2019025	99.44	0	1795
B2019024	100	0.33	1804

## Data Availability

The genomes data presented in this study are openly available in NCBI under BioProject accession PRJNA1298557.

## Supplementary Materials

The following supporting information can be downloaded at https://www.mdpi.com/article/10.3390/microorganisms14040874/s1. Table S1: The information of isolated strains. Table S2: Summary of the collection information of the reference genomes. Table S3: Significant mixed-effects model results for metadata-complete subset analyses assessing the robustness of major host-associated differences after accounting for geographic region and isolation year. Figure S1: Phylogenomic tree of dereplicated *C. jejuni* genomes based on single-copied Orthologs identified by OrthoFinder. Branches are colored according to isolation year; terminal symbols denote host sources (different shapes); genome labels are color-coded by continent of origin.
