# Supplementary material for: Cross-Host Adaptation of Campylobacter jejuni Is Shaped by Chromosomal Backgrounds and Mobile Gene Acquisition, with Human-Associated Traits Emerging Under Limited Mutational Diversification
Source: Microorganisms. 2026 Apr 13;14(4):874. doi: 10.3390/microorganisms14040874 (PMC13119460; doi:10.3390/microorganisms14040874)
Supplement: Supplementary file 1 [file microorganisms-14-00874-s001.zip › Supplymentary.pdf]

Cross-Host Adaptation of *Campylobacter jejuni* Is Shaped by Chromosomal Backgrounds and Mobile Gene Acquisition, with Human-Associated Traits Emerging Under Limited Mutational Diversification

Yingdong Li, Zhifeng Ma, Jing Chi, Yinqiu Wang, Mingjie Li, Qianru Wang, Lei Lei* and Qingliang Chen*

Department of Microbiological Laboratory, Baoan District Center for Disease Control and Prevention, Shenzhen 518101, China

***correspondence: leilei1980baoan@gmail.com (L.L.);**
15915333175@163.com (Q.C.)

Keywords:
Campylobacter jejuni; host adaptation; horizontal gene transfer; plasmids; zoonosis; comparative genomics

Table S1 The information of isolated strains

Genomes	Year	Location	Host
WQ2018012	2018	Shenzhen	Ducks
BWQ2018034	2018	Shenzhen	Birds
BWQ2018033	2018	Shenzhen	Ducks
BWQ2018032	2018	Shenzhen	Ducks
BWQ2018031	2018	Shenzhen	Ducks
BWQ2018030	2018	Shenzhen	Chicken
BWQ2018029	2018	Shenzhen	Ducks
BWQ2018028	2018	Shenzhen	Birds
BWQ2018027	2018	Shenzhen	Ducks
BWQ2018026	2018	Shenzhen	Ducks
BWQ2018025	2018	Shenzhen	Chicken
BWQ2018024	2018	Shenzhen	Chicken
BWQ2018023	2018	Shenzhen	Ducks
BWQ2018022	2018	Shenzhen	Birds
BWQ2018021	2018	Shenzhen	Ducks
BWQ2018020	2018	Shenzhen	Birds
BWQ2018019	2018	Shenzhen	Birds
BWQ2018017	2018	Shenzhen	Ducks
BWQ2018016	2018	Shenzhen	Birds
BWQ2018015	2018	Shenzhen	Chicken
BWQ2018014	2018	Shenzhen	Chicken
BWQ2018013	2018	Shenzhen	Ducks
BWQ2018012	2018	Shenzhen	Ducks
BWQ2018010	2018	Shenzhen	Birds
BWQ2018009	2018	Shenzhen	Ducks
BWQ2018008	2018	Shenzhen	Ducks
BWQ2018007	2018	Shenzhen	Chicken
BWQ2018006	2018	Shenzhen	Ducks
BWQ2018005	2018	Shenzhen	Chicken
BWQ2018003	2018	Shenzhen	Ducks
BWQ2018002	2018	Shenzhen	Human
BWQ2018001	2018	Shenzhen	Chicken
BWQ2017004	2017	Shenzhen	Birds
BWQ2017003	2017	Shenzhen	Birds
BWQ2017002	2017	Shenzhen	Birds
BWQ2017001	2017	Shenzhen	Birds
BWQ2016002	2016	Shenzhen	Ducks
BWQ2016001	2016	Shenzhen	Ducks
B2023021	2023	Shenzhen	Chicken
B2023023	2023	Shenzhen	Chicken
B2022033	2022	Shenzhen	Birds
B2022032	2022	Shenzhen	Birds

B2022031	2022	Shenzhen	Birds
B2022030	2022	Shenzhen	Birds
B2021152	2021	Shenzhen	Chicken
B2021019	2021	Shenzhen	Birds
B2021018	2021	Shenzhen	Chicken
B2021017	2021	Shenzhen	Birds
B2021024	2021	Shenzhen	Birds
B2021015	2021	Shenzhen	Birds
B2019099	2019	Shenzhen	Ducks
B2019098	2019	Shenzhen	Ducks
B2019097	2019	Shenzhen	Ducks
B2019096	2019	Shenzhen	Ducks
B2019044	2019	Shenzhen	Ducks
B2019043	2019	Shenzhen	Chicken
B2019029	2019	Shenzhen	Birds
B2019028	2019	Shenzhen	Chicken
B2019027	2019	Shenzhen	Chicken
B2019025	2019	Shenzhen	Chicken
B2019024	2019	Shenzhen	Birds

Table S3 Significant mixed-effects model results for metadata-complete subset analyses assessing the robustness of major host-associated differences after accounting for geographic region and isolation year

Response variable	Metadata-complete subset used (n)	Model formula	Fixed effect / contrast	Estimate (β)	95% CI of β	Adjusted q	Region variance	Year variance	Approx. post hoc power	Power interpretation
Coding density	284	Coding density ~ host + (1|region) + (1|year)	Human vs Mammal	-0.017	-0.028 to -0.007	0.009	0.0074	0.0064	0.78	Moderate-to-high
C-ARSC	284	C-ARSC ~ host + (1|region) + (1|year)	Human vs avian	0.018	0.008 to 0.028	0.006	0.0021	0.0008	0.96	High
C-ARSC	284	C-ARSC ~ host + (1|region) + (1|year)	Mammal vs avian	0.013	0.009 to 0.021	0.011	0.0017	0.0006	0.91	High
N-ARSC	284	N-ARSC ~ host + (1|region) + (1|year)	Human vs avian	0.014	0.005 to 0.021	0.011	0.0130	0.0410	0.83	Moderate-to-high
N-ARSC	284	N-ARSC ~ host + (1|region) + (1|year)	Mammal vs avian	0.012	0.007 to 0.023	0.019	0.0240	0.0210	0.76	Moderate
Enrichment ratio of host-associated unique genes in KEGG modules	284	Enrichment ratio ~ host + (1|region) + (1|year)	Human vs avian	0.018	0.007 to 0.027	0.004	0.0020	0.0006	0.87	Moderate-to-high

Response variable	Metadata-complete subset used (n)	Model formula	Fixed effect / contrast	Estimate (β)	95% CI of β	Adjusted q	Region variance	Year variance	Approx. post hoc power	Power interpretation
Enrichment ratio of host-associated unique genes in KEGG modules	284	Enrichment ratio ~ host + (1|region) + (1|year)	Mammal vs avian	0.022	0.013 to 0.031	0.031	0.0610	0.0140	0.69	Moderate
Enrichment ratio of host-associated unique genes in VFDB module	284	Enrichment ratio ~ host + (1|region) + (1|year)	Human vs avian	0.270	0.140 to 0.370	0.020	0.0340	0.0100	0.81	Moderate-to-high
Enrichment ratio of host-associated unique genes in VFDB module	284	Enrichment ratio ~ host + (1|region) + (1|year)	Mammal vs avian	0.100	0.082 to 0.183	0.031	0.0140	0.0005	0.73	Moderate
Chromosome-encoded key genes	284	Enrichment ratio ~ host + (1|region) + (1|year)	Human vs other host groups	0.130	0.072 to 0.240	0.034	0.0210	0.0017	0.71	Moderate
Plasmid- and HTG-associated key genes	284	Enrichment ratio ~ host + (1|region) + (1|year)	Human vs other host groups	0.160	0.074 to 0.250	0.037	0.0190	0.0190	0.68	Moderate

*Only contrasts that remained statistically significant after Benjamini–Hochberg correction (adjusted $q < 0.05$) are shown. Avian indicates birds, ducks, and chickens. Approximate post hoc power values were estimated from the fitted model contrasts and are provided as heuristic indicators of support strength rather than exact design-based power estimates.

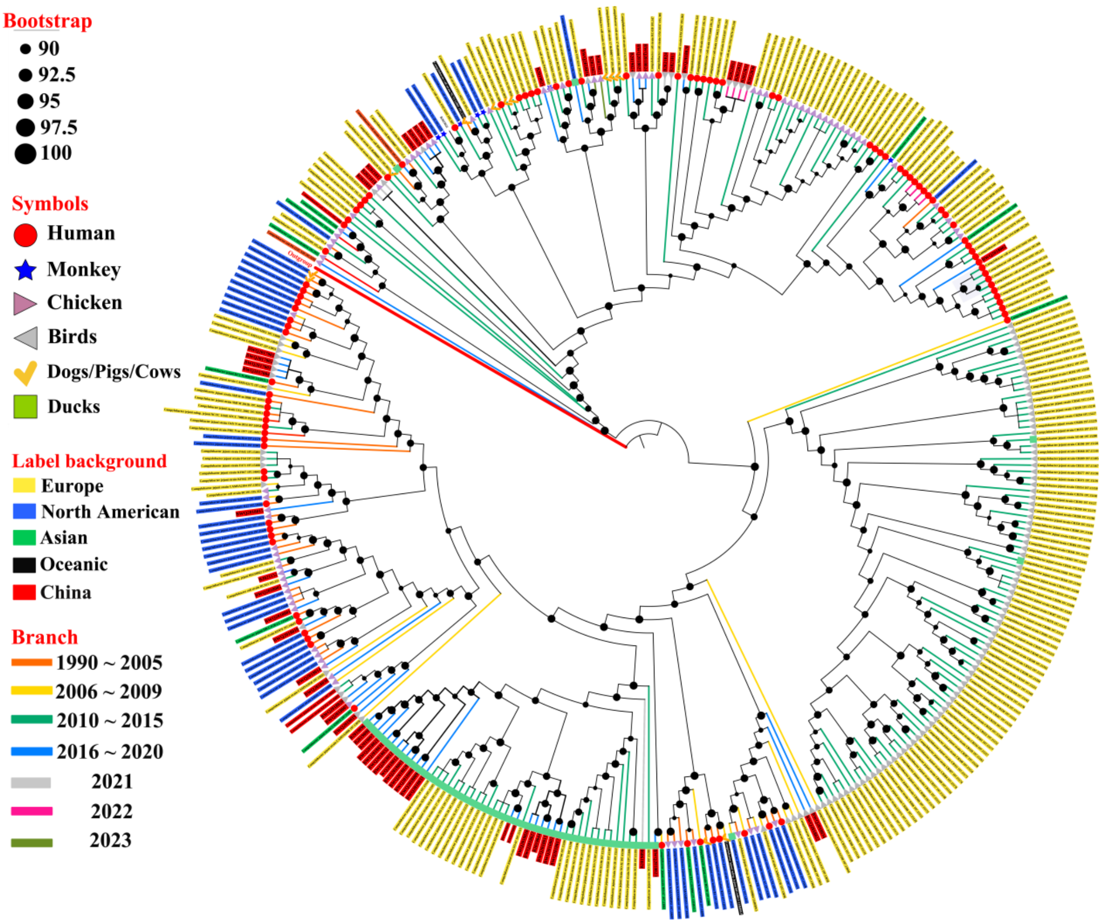

Figure S1. Phylogenomic tree of dereplicated *C. jejuni* genomes based on single-copied Orthologs identified by OrthoFinder. Branches are colored according to isolation year; terminal symbols denote host sources (different shapes); genome labels are color-coded by continent of origin.
